# Association of aortic valve calcification with carotid artery lesions and peripheral artery disease in patients with chronic kidney disease: a cross-sectional study

**DOI:** 10.1186/s12882-020-01864-z

**Published:** 2020-05-29

**Authors:** Yui Arita, Masaru Nakayama, Yuta Matsukuma, Ryota Yoshitomi, Makiko Seki, Akiko Fukui, Susumu Tsuda, Yuri Sonoda, Rina Imazu, Kimika Arakawa, Mitsuhiro Tominaga, Toshiaki Nakano, Kazuhiko Tsuruya, Takanari Kitazono

**Affiliations:** 1 Division of Nephrology, Department of Internal Medicine, Yamaguchi Red Cross Hospital, 53-1 Yawatanobaba, Yamaguchi, 753-0092 Japan; 2 Department of Internal Medicine, National Hospital Organization Kyushu Medical Center, Division of Nephrology and Clinical Research Institute, 1-8-1 Jigyohama, Chuo-ku, Fukuoka, 810-8563 Japan; 3 Department of Internal Medicine, National Hospital Organization Kyushu Medical Center, Division of Hypertension and Clinical Research Institute, 1-8-1 Jigyohama, Chuo-ku, Fukuoka, 810-8563 Japan; 4 Department of Medicine and Clinical Science, Graduate School of Medical Sciences, Kyushu University, 3-1-1 Maidashi, Higashi-ku, Fukuoka, 812-8582 Japan; 5 Department of Nephrology, Nara Medical University, 840 Shijo-cho, Kashihara, Nara, 634-8521 Japan

**Keywords:** Aortic valve calcification, Atherosclerosis, Carotid artery plaque, Chronic kidney disease, Peripheral artery disease

## Abstract

**Background:**

Patients with chronic kidney disease (CKD) reportedly have a high prevalence of aortic valve calcification (AVC). In population-based studies, AVC is considered a manifestation of systemic atherosclerosis. The association of AVC with atherosclerotic lesions has not been fully investigated in predialysis patients. The present study was performed to determine whether carotid artery lesions and peripheral artery disease (PAD) are associated with AVC in patients with CKD not on dialysis.

**Methods:**

In total, 749 patients were included in this cross-sectional study. AVC was evaluated using echocardiography. Carotid artery lesions including carotid artery plaque (CAP) and PAD were simultaneously examined in each patient. A logistic regression analysis was applied to determine the factors associated with AVC.

**Results:**

AVC, CAP, and PAD were found in 201, 583, and 123 patients, respectively. In the multivariable analyses adjusted for covariates including the estimated glomerular filtration rate and makers of mineral metabolism (serum calcium, serum phosphorus, parathyroid hormone, 1,25-dihydroxyvitamin D, and fibroblast growth factor 23), AVC was significantly associated with the presence of CAP [odds ratio (OR), 3.37; 95% confidence interval (CI), 1.43–7.95], the presence of PAD (OR, 1.76; 95% CI, 1.10–2.81), the CAP score (per 1.0-point increase) (OR, 1.06; 95% CI, 1.02–1.11), and the ankle-brachial blood pressure index (per 0.1-point increase) (OR, 0.83; 95% CI, 0.72–0.95).

**Conclusions:**

AVC was associated with atherosclerotic lesions independent of kidney function and mineral metabolism. We consider that this association between AVC and atherosclerosis might reflect the burden of shared atherosclerotic risk factors.

## Background

Patients with chronic kidney disease (CKD) are considered to have a high risk of cardiovascular (CV) morbidity and mortality [1]. A previous study showed that left-sided valve disease such as aortic stenosis and mitral regurgitation was highly prevalent and related to higher mortality in patients with CKD [2]. Mortality due to CV disease is 10–30-fold higher in dialysis patients than in the general population [3]. In addition, in predialysis patients, the risk of all-cause mortality increased as estimated glomerular filtration rate (eGFR) decreased, in one study, especially in patients with eGFR < 15 mL/min/1.73 m^2^, who had a 5.9-fold increase in the risk of all-cause mortality compared with that in patients with eGFR ≥60 mL/min/1.73 m^2^ [4]. Research has demonstrated that moderate/severe aortic valve calcification (AVC) is associated with CV events and mortality in patients with aortic stenosis [5, 6] and in the general population [7, 8]. Furthermore, in dialysis [9] or predialysis [10] patients, univariable analysis showed a significant association of AVC with all-cause mortality; however, multivariable analysis did not.

The reported prevalence of AVC ranges from 6.6 to 18.4% in population-based studies [7, 8, 11–15]**.** In contrast, patients with CKD have been shown to have a high prevalence of AVC. In dialysis patients, the prevalence of AVC ranges from 14.1 to 75.0% [9, 16–19], while predialysis patients with CKD show a prevalence of 18.6 to 36.5% [10, 20–22]. Many studies to date have explored the factors associated with AVC. Traditional CV risk factors such as hypertension, diabetes mellitus, smoking, dyslipidemia, male sex, and older age, similar to the risk factors for atherosclerosis, have been associated with AVC in population-based studies [14, 23]. In patients with CKD, Leskinen et al. [24] and Wang et al. [17] demonstrated that a combination of aortic and mitral valve calcification was associated with carotid artery lesions including carotid artery plaque (CAP) and peripheral artery disease (PAD), which are recognized as surrogate markers of subclinical atherosclerosis [25, 26]. The study participants were predialysis, dialysis, and renal transplantation patients in the former report and dialysis patients in the latter report. In addition, among dialysis patients, one study showed that the carotid intima-media thickness (IMT) was significantly higher in patients with than without AVC [19]. Nevertheless, whether an association of AVC with carotid artery lesions and PAD exists in predialysis patients remains unknown. Furthermore, especially in patients with CKD, in addition to traditional CV risk factors, nontraditional CV risk factors such as renal dysfunction itself and deranged mineral metabolism [e.g., hyperphosphatemia, elevated parathyroid hormone (PTH) or fibroblast growth factor 23 (FGF23)] associated with CKD are also associated with AVC [27–31]. However, in predialysis patients, the association of AVC with carotid artery lesions and PAD beyond both traditional and nontraditional CV risk factors remains uncertain. Thus, we performed the present study to elucidate whether both carotid artery lesions and PAD are associated with AVC independent of traditional and nontraditional CV risk factors associated with CKD in patients with stage 1–5 CKD not receiving dialysis.

## Methods

### Patients and study design

In total, 821 patients were admitted to our hospital for evaluation and education of CKD from May 2010 to July 2019. We excluded 72 patients who underwent aortic valve replacement (*n* = 7) or had no available data regarding echocardiography (*n* = 7), blood samples (*n* = 33), ankle-brachial blood pressure index (ABPI) (*n* = 5), or carotid artery lesions (*n* = 20). The remaining 749 patients were enrolled in this cross-sectional study, and no patients had bicuspid aortic valves or rheumatic heart disease.

Blood samples were obtained from each patient in the early morning after an overnight fast. Daily proteinuria was also measured. The intact FGF23 level was measured using an FGF23 enzyme-linked immunosorbent assay kit (Kainos Laboratories Inc., Tokyo, Japan). The intact PTH level was measured by electrochemiluminescence immunoassay (Elecsys PTH; Roche Diagnostics GmbH, Mannheim, Germany). A radioimmunoassay was used to measure the 1,25-dihydroxyvitamin D [1,25(OH)_2_D] level (Immunodiagnostic Systems Limited, Boldon, UK). The eGFR (mL/min/1.73 m^2^) was calculated using the new Japanese equation: eGFR = 194 × SCr^− 1.094^ × age^− 0.287^ × 0.739 (if female) [32], where SCr is the serum creatinine level. CKD was defined as abnormal kidney structure or function that persisted for > 3 months, as indicated by eGFR < 60 mL/min/1.73 m^2^ or by kidney damage regardless of a decrease in eGFR, in accordance with the Kidney Disease: Improving Global Outcomes clinical practice guidelines [33].

All enrolled patients were interviewed and clinically examined at presentation. Their medical histories and outpatient records were evaluated in detail. Demographic information (age and sex), medication history, and atherosclerotic risk factors (hypertension, history of smoking, dyslipidemia and diabetes mellitus) at presentation were recorded for each patient. Hypertension was defined as systolic blood pressure of ≥140 mmHg, diastolic blood pressure of ≥90 mmHg, or the current use of antihypertensive drugs. Diabetes mellitus was defined as a previous or current plasma fasting glucose level of ≥6.99 mmol/L or the use of hypoglycemic agents. Dyslipidemia was defined as plasma triglycerides ≥1.69 mmol/L, plasma low-density lipoprotein cholesterol ≥3.62 mmol/L, plasma high-density lipoprotein cholesterol < 1.03 mmol/L, or the use of lipid-lowering drugs based on a history of dyslipidemia. Ischemic heart disease was defined as a history of angina, myocardial infarction, coronary angioplasty, or coronary artery bypass surgery. Cigarette smoking was evaluated as current or past. The body mass index was calculated as weight in kg divided by height in m^2^. Blood pressure was measured on the second day of hospitalization at three separate times, with the patient in a sitting position; the average of the three readings was recorded.

The method of ABPI measurement is described in our previous report [34]. PAD was defined as having a low ABPI (< 0.9) [35] or undergoing interventions for lower limb ischemia. In addition, we examined the carotid IMT using a 5- to 12-MHz linear array transducer with a high-resolution B-mode scanning protocol, as previously described [36]. The CAP score was calculated by summing the thickness of all plaques in the bilateral carotid bifurcation and the bilateral common and internal carotid arteries. Echocardiography was performed simultaneously in each patient. The definition of left ventricular hypertrophy is described in our previous report [37]. The presence or absence of AVC was determined visually and only qualitatively using echocardiography, and the number of calcified aortic cusps was examined.

### Statistical analysis

Continuous data are expressed as either mean ± SD or median (interquartile range), depending on their distribution. Categorical data are expressed as n (%). The non-normally distributed C-reactive protein, FGF23, PTH, and 1,25(OH)_2_D levels were log-transformed to achieve an approximately normal distribution prior to statistical analysis. A logistic regression model was also applied to elucidate the factors associated with AVC. The odds ratio (OR) and 95% confidence interval (CI) were calculated for each variable. In addition, we examined linear regression analyses to evaluate the association between AVC score and CAP score, ABPI, and eGFR. All statistical analyses were performed using STATA ver. 14 (StataCorp. 2015. Stata Statistical Software. Release 14. College Station, TX: StataCorp LP.). A *P-*value of < 0.05 was considered to indicate statistical significance.

## Results

The median age of the 749 patients (499 men and 250 women) in this study was 70 years (range, 20–95 years). The primary causes of renal disease were chronic glomerulonephritis (29.1%, 218 patients), hypertensive nephrosclerosis (28.7%, 215 patients), diabetic nephropathy (21.9%, 164 patients), other defined causes (17.5%, 131 patients), and unknown causes (2.8%, 21 patients). Of the total 749 patients, 127 (17.0%), 221 (29.5%), 237 (31.6%), and 164 (21.9%) patients were categorized as having stage 1–2, 3, 4, and 5 CKD, respectively. The prevalence of AVC was 26.8% (201 patients). CAP and PAD were found in 583 and 123 patients, respectively. The prevalence of AVC in patients with stage 1–2, 3, 4, and 5 CKD was 11.0, 25.3, 30.8, and 35.4%, respectively (*P* for trend < 0.01). Table 1 shows the clinical characteristics of patients with and without AVC. Patients with AVC were divided into three groups according to the number of calcified aortic cusps. Patients of older ages experienced a higher prevalence of AVC. In patients with AVC, the prevalence of hypertension, diabetes mellitus, ischemic heart disease, and left ventricular hypertrophy was significantly higher while the eGFR and serum albumin level were significantly lower than those in patients without AVC. Pack-years of smoking was higher in patients with AVC. With regard to the association of AVC with mineral metabolism, a lower 1,25(OH)_2_D level and a higher FGF23 level were related to AVC. AVC was significantly associated with the presence of CAP and PAD, a higher CAP score, and a lower ABPI. In addition, linear regression analyses showed that AVC score was related to CAP score (supplementary material 1), ABPI (supplementary material 2), and eGFR (supplementary material 3).

**Table 1:** Clinical characteristics of patients with and without AVC

Variables	All patients	AVC (−)	AVC (+) (*n* = 201)			^a^*P* for trend
			1 aortic cusp	2 aortic cusps	3 aortic cusps	
	(*n* = 749)	(*n* = 548)	(*n* = 116)	(*n* = 47)	(*n* = 38)	
Age (years)	70 (59–78)	66 (53–75)	76 (69–80)	80 (74–84)	80 (74–83)	< 0.01
Male, n (%)	499 (67)	357 (65)	80 (69)	35 (74)	27 (71)	0.15
Hypertension, n (%)	617 (82)	433 (79)	102 (88)	44 (94)	38 (100)	< 0.01
Diabetes mellitus, n (%)	283 (38)	191 (35)	51 (44)	24 (51)	17 (45)	0.01
Smoking, n (%)	406 (54)	287 (52)	68 (59)	29 (62)	22 (58)	0.14
Pack-years of smoking^b^	6 (0–40)	2 (0–37)	20 (0–48)	30 (0–50)	9 (0–56)	< 0.01
IHD, n (%)	109 (15)	63 (12)	21 (18)	8 (17)	17 (45)	< 0.01
Dyslipidemia, n (%)	552 (74)	404 (74)	88 (76)	33 (70)	27 (71)	0.70
SBP (mmHg)	133 ± 18	132 ± 18	136 ± 17	136 ± 19	135 ± 17	< 0.01
DBP (mmHg)	72 (65–80)	73 (66–81)	71 (65–79)	67 (60–75)	68 (64–76)	< 0.01
Use of statins, n (%)	266 (36)	177 (32)	55 (47)	16 (34)	18 (47)	0.02
Use of active vitamin D_3,_ n (%)	55 (7)	30 (5)	17 (15)	2 (4)	6 (16)	0.01
Body mass index (kg/m^2^)	22.9 (20.5–25.1)	22.9 (20.6–25.5)	23.0 (20.7–24.7)	23.0 (19.8–25.3)	22.2 (20.9–24.0)	0.28
Daily proteinuria (g)	0.95 (0.28–2.63)	0.88 (0.26–2.59)	1.06 (0.30–2.59)	1.44 (0.57–3.28)	0.73 (0.21–2.54)	0.62
CRP (mg/L)	1.0 (0.5–2.0)	0.9 (0.5–2.0)	0.9 (0.5–2.0)	1.2 (0.6–2.0)	1.3 (0.5–2.9)	0.16
eGFR (mL/min/1.73m^2^)	27.9 (16.0–47.6)	30.4 (17.2–53.1)	23.0 (14.1–37.1)	25.8 (14.7–36.8)	22.9 (12.9–31.7)	< 0.01
HDL-C (mmol/L)	1.16 (0.93–1.45)	1.16 (0.93–1.45)	1.12 (0.96–1.42)	1.16 (0.93–1.42)	1.22 (0.91–1.55)	0.84
LDL-C (mmol/L)	2.51 (1.99–3.10)	2.53 (2.04–3.15)	2.38 (1.95–3.00)	2.53 (1.99–3.10)	2.20 (1.81–2.92)	0.05
Serum albumin (g/L)	34 (30–38)	35 (31–38)	34 (29–36)	31 (25–35)	33 (30–36)	< 0.01
Serum calcium (mmol/L)	2.32 (2.25–2.40)	2.32 (2.25–2.37)	2.32 (2.27–2.42)	2.32 (2.25–2.47)	2.30 (2.25–2.40)	0.13
Serum phosphorus (mmol/L)	1.19 (1.07–1.32)	1.19 (1.07–1.32)	1.19 (1.07–1.36)	1.16 (1.07–1.29)	1.26 (1.13–1.39)	0.28
PTH (pg/mL)	62 (43–102)	60 (40–100)	64 (43–107)	74 (55–116)	65 (43–102)	0.24
1,25(OH)_2_D (pg/mL)	28.9 (20.4–39.6)	30.2 (21.2–40.5)	25.2 (18.6–35.5)	29.0 (20.4–37.7)	24.1 (16.7–35.8)	0.01
FGF23 (pg/mL)	83 (57–130)	79 (54–125)	89 (60–152)	89 (70–132)	102 (71–158)	< 0.01
LVH, n (%)	338 (45)	222 (41)	68 (59)	25 (53)	23 (61)	< 0.01
Carotid IMT (mm)	0.70 (0.60–0.82)	0.68 (0.58–0.80)	0.75 (0.66–0.87)	0.75 (0.69–0.83)	0.76 (0.67–0.86)	< 0.01
CAP, n (%)	583 (78)	389 (71)	113 (97)	46 (98)	35 (92)	< 0.01
CAP score	4.49 (1.28–8.46)	3.40 (0.00–7.00)	7.10 (3.54–10.96)	7.41 (3.57–12.46)	8.90 (6.30–12.19)	< 0.01
PAD, n (%)	123 (16)	65 (12)	28 (24)	15 (32)	15 (39)	< 0.01
ABPI	1.07 (1.00–1.14)	1.08 (1.02–1.15)	1.05 (0.95–1.13)	1.07 (0.90–1.16)	0.95 (0.89–1.08)	< 0.01

Values are expressed as mean ± SD, n (%) or median (interquartile range). *AVC* aortic valve calcification, *IHD* ischemic heart disease, *SBP* systolic blood pressure, *DBP* diastolic blood pressure, *CRP* C-reactive protein, *eGFR* estimated glomerular filtration rate, *HDL-C* high-density lipoprotein cholesterol, *LDL-C* low-density lipoprotein cholesterol, *PTH* parathyroid hormone, *1,25(OH)*_*2*_*D* 1,25-dihydroxyvitamin D, *FGF23* fibroblast growth factor 23, *LVH* left ventricular hypertrophy, *IMT* intima-media thickness, *CAP* carotid artery plaque, *PAD* peripheral artery disease, *ABPI* ankle-brachial blood pressure index. ^a^Trend analyses were performed across 4 groups (1 group without AVC and 3 groups with AVC). ^b^Ten patients had no available data

The univariable logistic regression analyses are shown in Table 2. Older age, higher systolic blood pressure, and the presence of diabetes mellitus and ischemic heart disease were significantly associated with AVC. The use of statins and active vitamin D_3_ was associated with AVC. A lower eGFR and serum albumin level were related to AVC. As for the association between AVC and mineral metabolism, a higher serum calcium, lower log 1,25(OH)_2_D, and higher log FGF23 were related to AVC, whereas serum phosphorus and log PTH were not. Left ventricular hypertrophy was also associated with AVC. In addition, patients with CAP, PAD, higher CAP score, lower ABPI, and higher carotid IMT had a significantly higher risk of having AVC. We performed multivariable analyses to determine whether the presence of CAP, CAP score, the presence of PAD, ABPI, and carotid IMT were independently associated with AVC (Table 3). Adjusted for age and sex (model 1), the presence of CAP, higher CAP score, the presence of PAD, and a lower ABPI were all associated with AVC. Conversely, we found no significant association between AVC and carotid IMT. Similarly, in the fully adjusted model (model 4), the presence of CAP, higher CAP score, the presence of PAD, and a lower ABPI, but not carotid IMT, were independent risk factors for AVC.

**Table 2:** Odds ratios for having AVC in the univariable analysis

Variables	OR	95% CI	*P*
Age (per 10-year increase)	2.49	2.06–3.01	< 0.01
Male	1.29	0.91–1.83	0.16
Diabetes mellitus	1.58	1.14–2.19	< 0.01
Smoking	1.32	0.95–1.83	0.10
Dyslipidemia	1.00	0.69–1.44	0.98
IHD	2.28	1.50–3.48	< 0.01
SBP (per 10-mmHg increase)	1.14	1.04–1.25	< 0.01
Body mass index (per 1-kg/m^2^ increase)	0.99	0.95–1.02	0.46
Log CRP (per 1-log unit increase)	1.12	0.97–1.30	0.12
Daily proteinuria (per 1-g increase)	1.00	0.95–1.06	0.92
eGFR (per 1-mL/min/1.73 m^2^ increase)	0.98	0.97–0.99	< 0.01
Serum albumin (per 1-g/L increase)	0.95	0.93–0.97	< 0.01
Serum calcium (per 1-mmol/L increase)	4.36	1.21–15.71	0.02
Serum phosphorus (per 1-mmol/L increase)	1.54	0.77–3.07	0.22
Log PTH (per 1-log unit increase)	1.04	0.83–1.31	0.71
Log 1,25(OH)_2_D (per 1-log unit increase)	0.61	0.44–0.85	< 0.01
Log FGF23 (per 1-log unit increase)	1.44	1.18–1.77	< 0.01
Use of statins	1.67	1.20–2.32	< 0.01
Use of active vitamin D_3_	2.45	1.40–4.28	< 0.01
LVH	2.00	1.44–2.78	< 0.01
CAP	11.33	5.21–24.62	< 0.01
CAP score (per 1.0-point increase)	1.15	1.12–1.20	< 0.01
PAD	3.01	2.02–4.50	< 0.01
ABPI (per 0.1-point increase)	0.71	0.63–0.80	< 0.01
Carotid IMT (per 0.1-mm increase)	1.35	1.22–1.49	< 0.01

*AVC* aortic valve calcification, *OR* odds ratio, *CI* confidence interval, *IHD* ischemic heart disease, *SBP* systolic blood pressure, *CRP* C-reactive protein, *eGFR* estimated glomerular filtration rate, *PTH* parathyroid hormone, *1,25(OH)*_*2*_*D* 1,25-dihydroxyvitamin D, *FGF23* fibroblast growth factor 23, *LVH* left ventricular hypertrophy, *CAP* carotid artery plaque, *PAD* peripheral artery disease, *ABPI* ankle-brachial blood pressure index, *IMT* intima-media thickness

**Table 3:** Odds ratios for having AVC in the multivariable analyses

	Model 1			Model 2			Model 3			Model 4		
	OR	95% CI	*P*	OR	95% CI	*P*	OR	95% CI	*P*	OR	95% CI	*P*
CAP	4.26	1.87–9.69	< 0.01	3.67	1.60–8.43	< 0.01	3.47	1.49–8.10	< 0.01	3.37	1.43–7.95	< 0.01
CAP score (per 1.0 increase)	1.08	1.04–1.13	< 0.01	1.07	1.03–1.12	< 0.01	1.09	1.02–1.11	< 0.01	1.06	1.02–1.11	< 0.01
PAD	1.95	1.26–3.01	< 0.01	1.73	1.10–2.71	0.02	1.77	1.12–2.80	0.02	1.76	1.10–2.81	0.02
ABPI (per 0.1 increase)	0.80	0.70–0.91	< 0.01	0.82	0.72–0.93	< 0.01	0.83	0.72–0.95	< 0.01	0.83	0.72–0.95	< 0.01
Carotid IMT (per 0.1-mm increase)	1.07	0.95–1.21	0.25	1.05	0.93–1.19	0.43	1.03	0.91–1.17	0.62	1.02	0.89–1.16	0.80

Model 1: adjusted for age and sex

Model 2: adjusted for model 1 plus smoking, diabetes mellitus, dyslipidemia, and systolic blood pressure

Model 3: adjusted for model 2 plus daily proteinuria, eGFR, serum albumin, serum calcium, serum phosphorus, log PTH, log 1,25(OH)_2_D, and log FGF23

Model 4: adjusted for model 3 plus the presence of ischemic heart disease, body mass index, log CRP, use of statins, use of active vitamin D_3_ and LVH

*AVC* aortic valve calcification, *OR* odds ratio, *CI* confidence interval, *CAP* carotid artery plaque, *PAD* peripheral artery disease, *ABPI* ankle-brachial blood pressure index, *IMT* intima-media thickness, *eGFR* estimated glomerular filtration rate, *PTH* parathyroid hormone, *1,25(OH)*_*2*_*D* 1,25-dihydroxyvitamin D, *FGF23* fibroblast growth factor 23, *CRP* C-reactive protein, *LVH* left ventricular hypertrophy

We also investigated the adjusted ORs for having AVC according to subgroups stratified by demographic and clinical characteristics, as shown in Table 4. In male patients and patients without diabetes mellitus, the presence of CAP and PAD, the CAP score, and the ABPI were all associated with AVC. Conversely, none of the above four variables showed significant associations with AVC in female patients and patients with diabetes mellitus. With regard to interactions for having AVC between those four variables and the other baseline clinical characteristics, the *P* values between CAP and the eGFR and among PAD, the eGFR, and the serum albumin level were < 0.1; in contrast, the other interactions showed *P* values of > 0.1.

**Table 4:** Adjusted odds ratios of CAP, CAP score, PAD, and ABPI for having AVC in subgroups stratified according to baseline characteristics

				CAP			CAP score (per 1.0-point increase)			PAD			ABPI (per 0.1-point increase)		
variables		No. of patients	No. of AVC	OR	95% CI	*P* for interaction	OR	95% CI	*P* for interaction	OR	95% CI	*P* for interaction	OR	95% CI	*P* for interaction
Age (years)	L (< 69.8)	375	44	16.79	^a^2.07–136.4	0.23	1.02	0.94–1.12	0.16	2.72	0.91–8.15	0.91	0.90	0.67–1.20	0.17
	H (> 69.8)	374	157	1.94	0.70–5.37		1.09	^a^1.03–1.15		1.84	^b^1.07–3.15		0.78	^a^0.66–0.92	
Sex	Male	499	142	3.55	^b^1.00–12.52	0.96	1.07	^a^1.02–1.13	0.82	2.04	^b^1.17–3.54	0.58	0.79	^a^0.67–0.93	0.49
	Female	250	59	3.12	0.93–10.39		1.06	0.97–1.17		1.04	0.36–3.01		0.96	0.70–1.30	
eGFR (mL/min/1.73m^2^)	L (< 28.0)	375	126	1.26	0.43–3.72	0.06	1.06	^b^1.00–1.12	0.40	1.62	0.90–2.93	0.049	0.78	^a^0.65–0.92	0.73
	H (> 28.0)	374	75	7.76	^a^1.70–35.47		1.08	0.999–1.17		1.94	0.78–4.77		0.91	0.69–1.20	
DM	Absence	466	109	4.91	^a^1.57–15.34	0.11	1.10	^a^1.03–1.17	0.09	2.17	^b^1.03–4.58	0.48	0.74	^b^0.58–0.94	0.27
	Presence	283	92	1.31	0.32–5.44		1.03	0.96–1.11		1.41	0.72–2.76		0.89	0.74–1.07	
S-Alb (g/L)	L (≤34)	383	128	4.71	^b^1.27–17.47	0.13	1.06	^b^1.00–1.12	0.21	2.45	^a^1.30–4.60	0.08	0.81	^b^0.67–0.97	0.62
	H (> 34)	366	73	1.54	0.46–5.24		1.12	^b^1.02–1.22		1.04	0.44–2.48		0.85	0.65–1.12	

*CAP* carotid artery plaque, *PAD* peripheral artery disease, *ABPI* ankle-brachial blood pressure index, *AVC* aortic valve calcification, *OR* odds ratio, *CI* confidence interval, *L* low, *H* high, *eGFR* estimated glomerular filtration rate, *DM* diabetes mellitus, *S-Alb* serum albumin. Adjusted for the same covariates in Model 4 in Table 3. ^a^*P* < 0.01; ^b^*P* < 0.05

## Discussion

The present study explored the factors associated with AVC in overall patients with CKD not on dialysis. The prevalence of AVC in our cohort was similar to that in previous reports [10, 22]. In the multivariable analysis, the presence of CAP and PAD, the CAP score, and ABPI were independent risk factors for AVC beyond traditional and nontraditional CV risk factors. In addition, the stratified analyses demonstrated that AVC was independently associated with any of the four variables of CAP, PAD, the CAP score, or the ABPI in all subgroups other than female patients and patients with diabetes mellitus.

The pathological mechanisms for the development of AVC are now believed to constitute an active cellular process that develops within the aortic valve leaflets, following mechanisms similar to atherosclerosis with lipoprotein deposition, chronic inflammation, and active leaflet calcification [27–29]. A previous population-based study showed that AVC was associated with a high incidence of atherosclerotic risk factors, suggesting that AVC is a manifestation of systemic atherosclerosis [14]. In patients with CKD, including dialysis patients, combined aortic and mitral valve calcification has also been regarded as a marker of the systemic atherosclerotic process [17, 24]. The present study was conducted in patients with CKD not on dialysis and also showed independent associations between AVC and subclinical atherosclerotic lesions such as CAP and PAD. These findings might suggest that AVC and atherosclerosis share common pathological mechanism and/or risk factors.

Several studies have addressed the association between kidney function and the prevalence of AVC. However, this relationship has remained unclear. A previous report showed a significant association of AVC with the presence of CKD; predialysis patients with CKD were approximately three times more likely to have AVC than patients without CKD [20]. In contrast, a large cohort study showed no significant association between the presence of CKD and the prevalence of AVC [38]. Another large cohort study also failed to show a significant association of AVC with impaired kidney function in the multivariable analysis [15]. The Chronic Renal Insufficiency Cohort (CRIC) Study demonstrated that decreased kidney function was significantly associated with AVC in the multivariable analysis after adjustment for traditional CV risk factors, but the relationship disappeared after adding nontraditional CV risk factors including high sensitivity C-reactive protein and plasma homocysteine as covariates [39]. In our cohort, the prevalence of AVC significantly increased with advancing CKD stages, and AVC was related to the eGFR in the univariable analysis; however, the multivariable analysis (model 4) did not show an association between AVC and the eGFR (data not shown). Furthermore, Piers et al. [21] noted that AVC was more frequent and severe in patients with atherosclerotic causes of CKD than in those with non-atherosclerotic causes of CKD, suggesting that AVC is associated with the atherosclerotic burden rather than the severity of CKD.

The association between AVC and derangement of mineral metabolism has been documented in several studies. Deranged mineral metabolism occurs with advancing CKD stages. One study showed that AVC was associated with higher FGF23 and PTH levels in patients with mild to moderate CKD [40], whereas a different population-based study showed no significant association of AVC with FGF23 or PTH [13]. In dialysis patients, the calcium × phosphate product has been shown to be a significant risk factor for AVC [41]. In population-based studies, the serum phosphorus level was also positively associated with the prevalence and incidence of AVC [12, 13]. Conversely, each 0.32-mmol/L increment in the serum phosphorus level in patients with CKD was associated with coronary artery and mitral valve calcification, but not with AVC [22]. Furthermore, the CRIC Study revealed that the serum calcium, serum phosphorus, and PTH levels were not independently associated with AVC [39]. The present study also showed significant associations of AVC with markers of mineral metabolism (serum calcium, log 1,25(OH)_2_D, and log FGF23) in the univariable analyses, but not in the multivariable analyses (model 4) (data not shown). Based on these findings, the substantial effects of derangement of mineral metabolism on the development of AVC have remained controversial, and further investigations are warranted to elucidate the association between AVC and deranged mineral metabolism.

In the present study, univariable logistic analysis revealed no significant association between AVC and male sex (Table 2). Furthermore, in the stratified analyses, we found no significant interactions between sex and parameters such as the presence of CAP, CAP score, the presence of PAD, and ABPI on AVC. However, each of these atherosclerotic parameters was associated with AVC in men, but not in women, although the reason for the lack of association of AVC with these parameters in women remains unclear.

### Study limitations

The present study has some limitations. First, all patients were recruited at a single regional hospital; thus, the selection of patients was limited and the sample size was relatively small. Second, our cohort had an imbalanced sex ratio and was relatively old, probably because of the recruitment of consecutive patients who were admitted to our hospital. Third, this cross-sectional study did not allow us to draw causal relationships. Fourth, the clinical data including the echocardiographic findings were recorded at one study time. Fifth, we did not evaluate fetuin-A, matrix-Gla-protein, reactive oxygen species, asymmetric dimethylarginine, or receptor activator of nuclear factor-κB ligand, all of which can play roles in aortic valve calcific degeneration associated with CKD [31]. Finally, we did not evaluate AVC quantitatively. AVC score quantitatively measured by computed tomography can detect valve calcification with higher sensitivity [42]; accordingly, the echocardiographic AVC evaluation method that we used is considered inferior to that with computed tomography. In this study, we performed multivariable logistic regression analyses, adjusted for several covariates, particularly markers of deranged mineral metabolism such as serum calcium, serum phosphorus, log PTH, log 1,25(OH)_2_D, and log FGF23, which were measured simultaneously. Accordingly, despite the limitations, our results may indicate a robust relationship between AVC and atherosclerotic parameters.

## Conclusions

The present study showed that AVC was associated with the presence of CAP and PAD, and the CAP score and ABPI, independent of traditional and nontraditional CV risk factors. We consider that the association between AVC and these subclinical atherosclerotic lesions might reflect the burden of shared atherosclerotic risk factors.

## Supplementary information

**Additional file 1: **Supplementary materials 1. Correlation between AVC score and CAP score. The dashed lines indicate 95% confidence intervals. *R*^2^ = 0.097, coefficient of correlation = 1.868, *P* < 0.01. AVC, aortic valve calcification; CAP, carotid artery plaque

**Additional file 2: **Supplementary materials 2. Correlation between AVC score and ABPI. The dashed lines indicate 95% confidence intervals. *R*^2^ = 0.048, coefficient of correlation = − 0.036, *P* < 0.01. AVC, aortic valve calcification; ABPI, ankle-brachial blood pressure index

**Additional file 3: **Supplementary materials 3. Correlation between AVC score and eGFR. The dashed lines indicate 95% confidence intervals. *R*^2^ = 0.027, coefficient of correlation = − 5.064, *P* < 0.01. AVC, aortic valve calcification; eGFR, estimated glomerular filtration rate

## Data Availability

The data used and analyzed in this study are available from the corresponding author on reasonable request.

## Abbreviations

CKD: Chronic kidney disease
CV: Cardiovascular
AVC: Aortic valve calcification
CAP: Carotid artery plaque
PAD: Peripheral artery disease
IMT: Intima-media thickness
ABPI: Ankle-brachial blood pressure index
FGF23: Fibroblast growth factor 23
PTH: Parathyroid hormone
1,25(OH)_2_D: 1,25-dihydroxyvitamin D
eGFR: Estimated glomerular filtration rate
OR: Odds ratio
CI: Confidence interval
CRIC: Chronic Renal Insufficiency Cohort
