# Supplementary figures and images for: Association of aortic valve calcification with carotid artery lesions and peripheral artery disease in patients with chronic kidney disease: a cross-sectional study
Source: BMC Nephrol. 2020 May 29;21:203. doi: 10.1186/s12882-020-01864-z (PMC7260754; doi:10.1186/s12882-020-01864-z)

## Slide 1
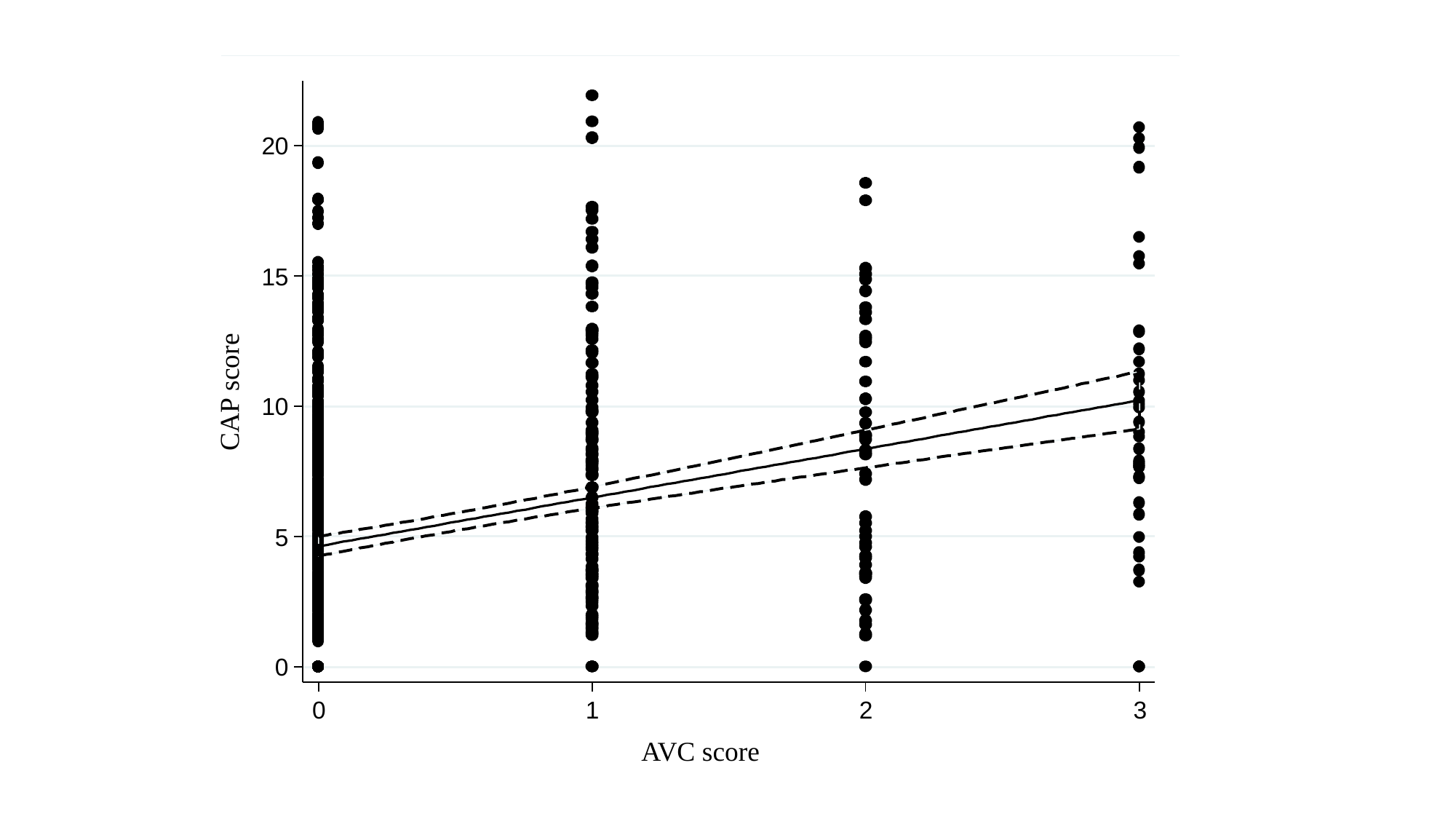

CAP score
AVC score

Supplement: Supplementary file 1 — Additional file 1: Supplementary materials 1. Correlation between AVC score and CAP score. The dashed lines indicate 95% confidence intervals. R2 = 0.097, coefficient of correlation = 1.868, P < 0.01. AVC, aortic valve calcification; CAP, carotid artery plaque [file 12882_2020_1864_MOESM1_ESM.pptx]

## Slide 1
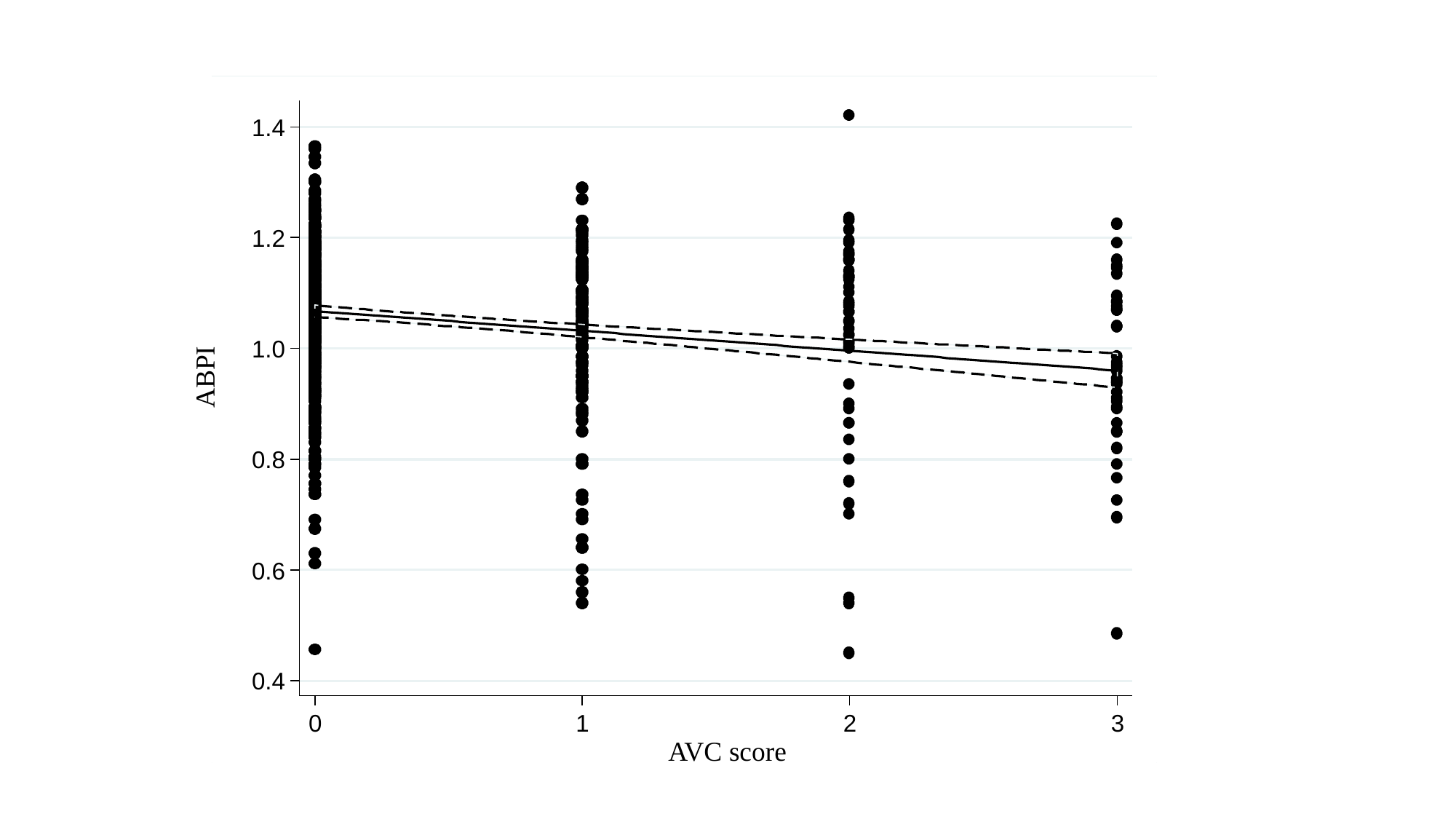

ABPI
AVC score

Supplement: Supplementary file 2 — Additional file 2: Supplementary materials 2. Correlation between AVC score and ABPI. The dashed lines indicate 95% confidence intervals. R2 = 0.048, coefficient of correlation = − 0.036, P < 0.01. AVC, aortic valve calcification; ABPI, ankle-brachial blood pressure index [file 12882_2020_1864_MOESM2_ESM.pptx]

## Slide 1
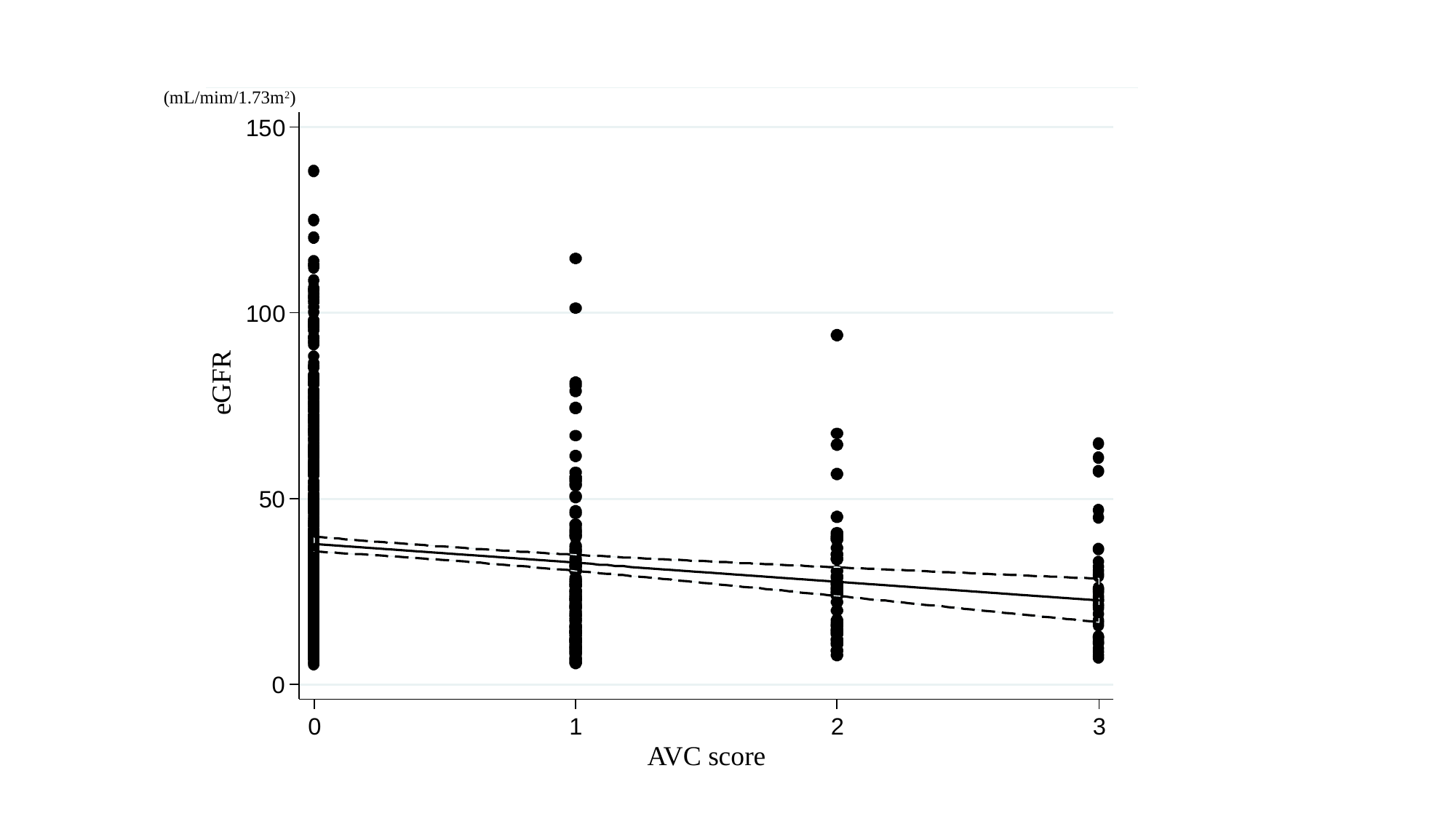

(mL/mim/1.73m2)
eGFR
AVC score

Supplement: Supplementary file 3 — Additional file 3: Supplementary materials 3. Correlation between AVC score and eGFR. The dashed lines indicate 95% confidence intervals. R2 = 0.027, coefficient of correlation = − 5.064, P < 0.01. AVC, aortic valve calcification; eGFR, estimated glomerular filtration rate [file 12882_2020_1864_MOESM3_ESM.pptx]
